# Generation, characterization and epitope mapping of monoclonal antibody 4H3 specific to the HN protein of pigeon paramyxovirus type 1 (PPMV-1)

**DOI:** 10.3389/fmicb.2026.1808407

**Published:** 2026-04-24

**Authors:** Xu Wang, Hongfeng Ren, Pei Li, Zhonglin Huang, Xing Li, Yang Wang, Yang Li, Libin Liang, Junping Li

**Affiliations:** 1 Shanxi Key Laboratory of Animal Disease Research, Prevention and Control, College of Veterinary Medicine, Shanxi Agricultural University, Jinzhong, China; 2 China Animal Health and Epidemiology Center, Qingdao, China

**Keywords:** antigenic epitope, application, HN protein, monoclonal antibody, pigeon paramyxovirus type 1 (PPMV-1)

## Abstract

Pigeon Newcastle disease (PND), caused by pigeon paramyxovirus type 1 (PPMV-1), is an acute, highly contagious disease that induces neurological symptoms and gastrointestinal disorders in pigeons, posing substantial economic risks to the global pigeon industry. The hemagglutinin-neuraminidase (HN) protein of PPMV-1 is a key surface glycoprotein mediating viral adsorption, invasion, and egress, and serves as a critical target for vaccine development and diagnostic reagent design. In this study, we aimed to generate a specific monoclonal antibody (mAb) against the HN protein of PPMV-1 and identify its recognized epitope. We codon-optimized the conserved 198–390 amino acid (aa) region of the HN protein from PPMV-1 strain, followed by prokaryotic expression and purification to serve as the immunogen. Through cell fusion monoclonal antibody screening, we successfully established a hybridoma cell line that secretes mAb 4H3. Western blot and immunofluorescence assay (IFA) confirmed that mAb 4H3 specifically reacts with PPMV-1 strains but exhibits no cross-reactivity with the commonly used NDV vaccine strain Lasota. Epitope mapping with a panel of truncated HN protein fragments identified the minimal B-cell epitope recognized by mAb 4H3 as ^310^DRVWF^314^. Sequence homology analysis showed this epitope is highly conserved across PPMV-1 strains. Collectively, our results offer a valuable tool for developing PPMV-1-specific diagnostic assays, as well as for investigations into HN protein function and the development of epitope-based vaccines against PND.

## 1 Introduction

Newcastle disease (ND), caused by virulent Newcastle disease virus (NDV, now reclassified as Avian Avulavirus 1, AAvV-1), is one of the most devastating poultry diseases worldwide, affecting a wide range of avian species and resulting in massive economic losses (Rehman et al., 2021; Wang et al., 2024). Pigeon paramyxovirus type 1 (PPMV-1), a genotype VI variant of NDV, was first isolated in the Middle East in the 1970s and has since become endemic globally (Shan et al., 2021; Xie et al., 2020; Goraichuk et al., 2023). Unlike classical NDV, PPMV-1 primarily infects pigeons and wild birds, causing pigeon Newcastle disease (PND) characterized by neurological symptoms (e.g., torticollis, wing deviation) and gastrointestinal disorders (Susta et al., 2016). Juvenile and suckling pigeons are highly susceptible to PPMV-1, with mortality rates ranging from 50% to 100% in severe cases (Cui et al., 2023; Zhang et al., 2025).

At present, the prevention and control of PND is primarily dependent on the administration of chicken Newcastle disease vaccines, yet the conventional Lasota strain of chicken NDV vaccine confers only limited protective efficacy against PPMV-1 (Nie et al., 2022; Wang et al., 2015; Jin et al., 2025). Additionally, rapid detection methodologies for PND are currently lacking. This highlights the urgent need for novel diagnostic tools and effective vaccines targeting PPMV-1 (Francisco et al., 2022). The HN protein of PPMV-1 is a multifunctional glycoprotein that plays pivotal roles in viral pathogenesis: it mediates binding to sialic acid receptors on host cells, enhances the fusion activity of the F protein, facilitates progeny virus release via neuraminidase activity, and acts as a major protective antigen inducing neutralizing antibodies (Zhao et al., 2014; Paldurai et al., 2014; Liu et al., 2019). Thus, the HN protein is an ideal target for the development of specific antibodies and epitope-based vaccines.

Monoclonal antibodies (mAbs) offer high specificity and uniformity, making them indispensable tools for viral diagnosis, antigen characterization, and vaccine evaluation (Martin et al., 2019; Guo et al., 2015; Zhang et al., 2022). In this study, we aimed to generate a mAb specific to the HN protein of PPMV-1, characterize its specificity, and identify its recognized epitope. The findings provide important support for the rapid detection, scientific prevention and control of PND.

## 2 Materials and methods

### 2.1 Viruses, cells, animals, and reagents

PPMV-1 strains (Pigeon/Shanxi/01, PG/SX/01; Pigeon/Jiangxi/812, PG/JX/812), chicken-derived NDV strains (Chicken/Shanxi/01, CK/SX/01; Chicken/Hunan/905, CK/HuN/905), duck-derived NDV strains (Duck/Guizhou/664, DK/GZ/664; Duck/Guangxi/463, DK/GX/463), NDV vaccine strain Lasota were preserved in our laboratory. Murine myeloma cells (SP2/0), human embryonic kidney cells (293 T), and DF-1 cells were maintained in our laboratory. SPF-grade BALB/c female mice (6–8 weeks old) were purchased from Vital River Laboratory (Beijing, China). IMDM medium, IMDM complete medium (supplemented with 15% fetal bovine serum), 2.3% methylcellulose, polyethylene glycol 1,450 (PEG1450), hypoxanthine-aminopterin-thymidine (HAT) medium, hypoxanthine-thymidine (HT) medium, and bovine serum albumin (BSA) were purchased from Sigma-Aldrich (St. Louis, MO, USA). Nickel-nitrilotriacetic acid (Ni-NTA) affinity chromatography columns were obtained from Beyotime Biotechnology (Shanghai, China). Goat anti-mouse IgG horseradish peroxidase (HRP)-conjugated secondary antibody and fluorescein isothiocyanate (FITC)-conjugated secondary antibody were purchased from Abcam (Cambridge, UK). *β*-actin and GAPDH antibody was obtained from Proteintech Group (Wuhan, China).

### 2.2 Codon optimization and prokaryotic expression of HN protein

Sequence alignment and secondary structure analysis were performed on the HN proteins of three PPMV-1 strains recently isolated and identified from clinical samples in our laboratory. Sequence alignment and secondary structure analysis revealed that the 198–390 amino acid (aa) region of the HN protein is highly conserved among PPMV-1 strains, with low hydrophobicity, high antigenicity, and favorable surface accessibility, rendering it an ideal candidate immunogen (Supplemental Figures S1–S3). The 198–390 aa region of the HN protein from PPMV-1 strain PG/SX/01 was selected as the target fragment. The coding sequence of this region was codon-optimized and cloned into the pET-30a expression vector via the *EcoRI* and *XhoI* restriction sites, and heterologously expressed in *Escherichia coli* (*E. coli*).

The recombinant plasmid pET-30a-HN_198–390_ was transformed into *E. coli* BL21 (DE3) competent cells. Positive clones were cultured at 37 °C to an OD_600_ of 0.6–0.8, then induced with 1 mM isopropyl *β*-D-thiogalactopyranoside (IPTG) at 37 °C for 8 h. Bacterial cells were harvested by centrifugation and sonicated for lysis. The cell lysate was centrifuged at 12,000 rpm for 20 min at 4 °C, and the resulting pellet was washed twice with washing buffer I (containing 1% Triton X-100 and 2 M urea) and once with washing buffer II (without detergents). Inclusion bodies were denatured in denaturation buffer (20 mM Tris–HCl, pH 8.0; 8 M urea; 10 mM dithiothreitol (DTT); 1 mM PMSF) at a concentration of 10 mg/mL for 2–4 h at room temperature. After centrifugation at 15,000 rpm for 30 min at 4 °C, the supernatant was subjected to gradient dialysis against buffers with gradually decreasing urea concentrations (4 M, 2 M, 0.5 M) at 4 °C. Renatured proteins were centrifuged to remove aggregates, loaded onto a pre-equilibrated Ni-NTA affinity column, washed with 20 mM imidazole to eliminate non-specific bindings, eluted with 250 mM imidazole, and dialyzed against PBS. SDS-PAGE verified the purity and molecular weight of the purified protein, and its concentration was quantified using a BCA protein assay kit.

### 2.3 Animal immunization and hybridoma cell preparation

Six Specific Pathogen Free (SPF) BALB/c mice were subcutaneously immunized with 60 μg of purified HN protein (198–390 aa) emulsified with Freund’s complete adjuvant (Sigma-Aldrich) for the primary immunization. Two booster immunizations were performed at 2-week intervals with 30 μg of the same protein emulsified with Freund’s incomplete adjuvant. Seven days after the second booster, mouse serum was collected to determine the antibody titer by indirect ELISA. Mice with serum titers >25,600 were selected for a final intraperitoneal booster immunization with 50 μg of purified HN protein (without adjuvant) 3 days before cell fusion.

Splenocytes were isolated from immunized mice and fused with SP2/0 myeloma cells by standard protocols. Hybridoma supernatants were screened for HN protein-specific antibodies via indirect ELISA. Positive clones were subcloned three times by limiting dilution to establish stable monoclonal hybridoma cell lines. A total of four positive hybridoma cell lines specific to the PPMV-1 HN protein were identified, including 4H3; among which, the hybridoma cell line secreting mAb 4H3 was subsequently selected for further characterization.

### 2.4 Subclass identification and titer determination of mAb 4H3

The subclass of mAb 4H3 was identified using a Mouse Monoclonal Antibody Isotyping Kit (Sigma-Aldrich) according to the manufacturer’s protocol. Briefly, 96-well plates were coated with goat anti-mouse Ig (Human ads-UNLB) and incubated with hybridoma supernatants. After washing, HRP-conjugated goat anti-mouse IgG1, IgG2a, IgG2b, IgG3, IgM, IgA, *κ*, and *λ* antibodies were added, followed by TMB color development. The absorbance was measured at 450 nm and 630 nm using a microplate reader (Bio-Tek, Winooski, VT, USA).

### 2.5 Binding specificity of mAb 4H3 to the HN proteins of different PPMV-1 and NDV strains

The binding specificity of mAb 4H3 to HN proteins of different origins was first determined by Western blot analysis. For this assay, DF-1 cells were infected with PPMV-1 (PG/SX/01, PG/JX/812), chicken-derived NDV (CK/SX/01, CK/HuN/905), duck-derived NDV (DK/GX/463, DK/GZ/664), or the commercial NDV Lasota vaccine strain at a multiplicity of infection (MOI) of 1. At 12 h post-infection (hpi), infected cells were lysed in NP-40 lysis buffer (Beyotime Biotechnology) supplemented with a protease inhibitor cocktail. Cell lysates were separated by SDS-PAGE and electrotransferred onto nitrocellulose (NC) membranes. After blocking with 5% skim milk at 37 °C for 1 h, the membranes were incubated with purified mAb 4H3 at 37 °C for 1 h, followed by incubation with HRP-conjugated goat anti-mouse IgG for an additional 1 h at the same temperature. Immunoreactive bands were visualized using an enhanced chemiluminescence (ECL) detection system (Bio-Rad, CA, USA).

Furthermore, the applicability of mAb 4H3 was assessed via immunofluorescence assay (IFA). For this assay, DF-1 cells were seeded into 12-well plates and infected with the aforementioned viruses at an Multiplicity of Infection (MOI) of 1. At 12 hpi, the cells were fixed with 4% paraformaldehyde for 30 min at room temperature, permeabilized with 0.5% Triton X-100 for 10 min, and blocked with 5% BSA at 37 °C for 1 h. The cells were then incubated with purified mAb 4H3 at room temperature for 1 h, followed by incubation with FITC-conjugated goat anti-mouse IgG for an additional 1 h in the dark. Cell nuclei were stained with 4′,6-diamidino-2-phenylindole (DAPI) for 5 min, and fluorescent signals were observed under a fluorescence microscope (Leica, Germany).

### 2.6 Epitope mapping of mAb 4H3

To identify the epitope recognized by mAb 4H3, a series of truncated HN protein fragments were constructed based on the 198–390 aa region. These fragments comprised HN_198–294_ and HN_295–390_ (the major binding regions of mAb 4H3 to the HN protein identified in the first round of epitope mapping); HN_295–330_, HN_295–350_ and HN_295–370_ (the critical binding subregions defined in the second round of screening); and a panel of N-terminally stepwise truncated mutants (5 amino acids per truncation) derived from HN_295–390_ (the third round of fine epitope mapping), including HN_300–390_, HN_305–390_, HN_310–390_, HN_315–390_, HN_320–390_ and HN_325–390_. Each truncated fragment was cloned into the pCAGGS-GST vector, and the recombinant plasmids were transfected into HEK293T cells using Lipofectamine LTX (Invitrogen, CA, USA). At 48 h post-transfection, the transfected cells were lysed, and the resulting cell lysates were subjected to Western blot analysis with mAb 4H3 to identify the specific truncated HN fragments recognized by this antibody.

### 2.7 Conservation analysis of the mAb 4H3-binding epitope

To compare the amino acid sequence differences of the HN protein at positions 310–314 (the critical epitope recognized by mAb 4H3) among PPMV-1, NDV, and NDV vaccine strains (including Lasota, Mukteswar, ZM10, and V4), SnapGene software and the online platform WeMol^1^ were used to visualize the sequence alignment results of the HN proteins from PPMV-1 and NDV, as well as the conservation of the amino acid sequence at positions 310–314. Protein models were constructed by referring to the previously reported NDV HN protein structure (PDB accession code: 3T1E). Subsequently, PyMOL was employed to visualize the localization of the epitope recognized by mAb 4H3 in the three-dimensional (3D) structure of the HN protein.

## 3 Results

### 3.1 Expression and purification of recombinant HN protein

**Figure 1:**
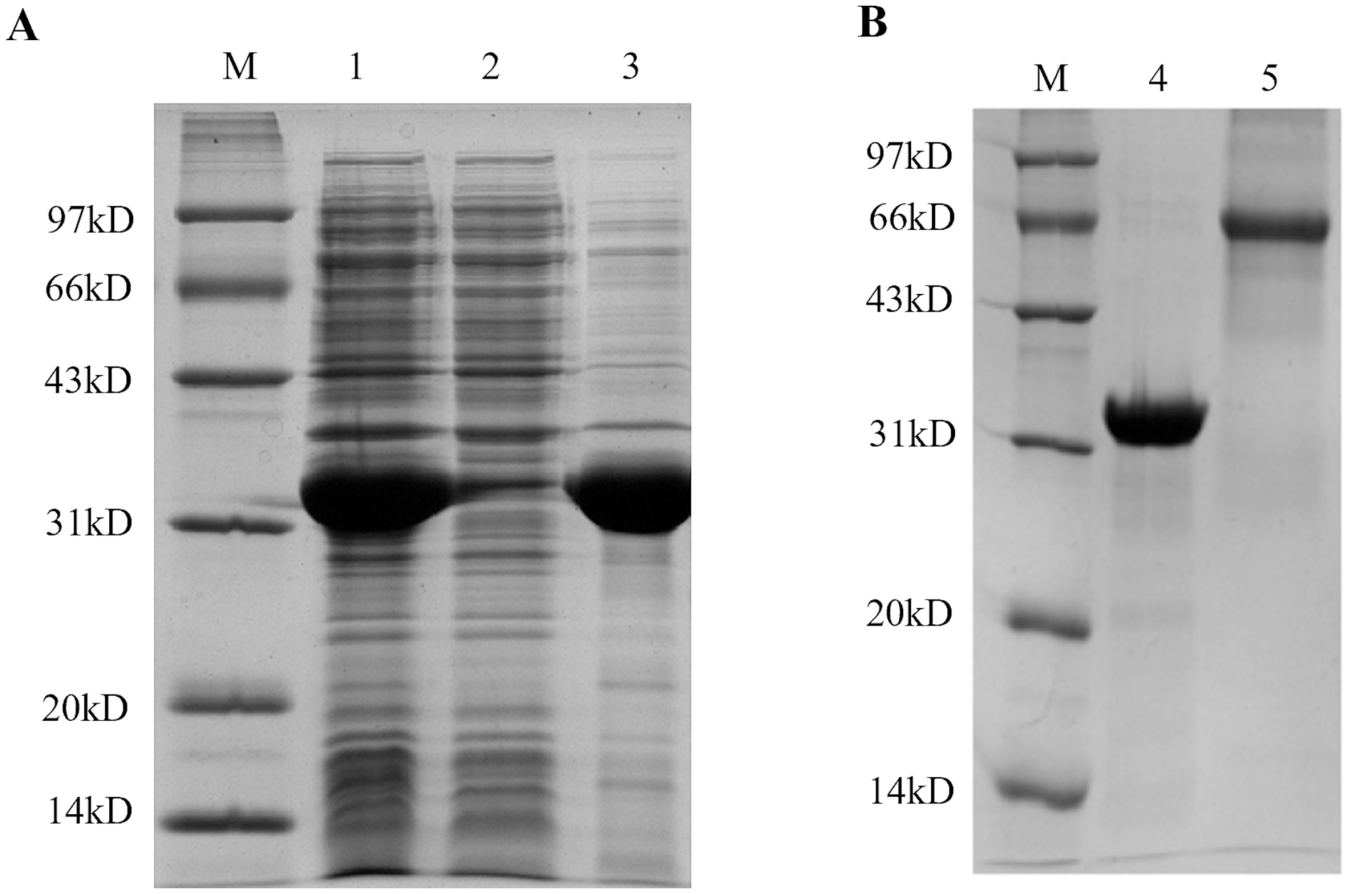
Expression and purification of the recombinant HN_198-390_ protein. **(A)** Prokaryotic expression of recombinant pET30a-HN_198-390_ fusion protein. Lane M, protein molecular weight marker; lanes 1–3, whole cell lysate, supernatant, and precipitate of IPTG-induced *E. coli* BL21 (DE3) transformed with pET30a-HN_198–390_, respectively. **(B)** Purification of recombinant His-tagged HN_198–390_ fusion protein. Lane M, protein molecular weight marker; lane 4, purified protein from tenfold dilution; lane 5, 0.5 mg/mL BSA (used as a loading control).

SDS-PAGE analysis showed that the recombinant HN protein (198–390 aa) was successfully expressed in *E. coli* BL21 (DE3) and purified by Ni-NTA chromatography. A single band with a molecular weight of approximately 33 kDa was observed, which was consistent with the expected size of the His-tagged HN fusion protein (Figure 1A). The purified protein exhibited high purity (>90%) as confirmed by SDS-PAGE (Figure 1B), and the concentration was adjusted to 1 mg/mL for subsequent experiments.

### 3.2 Subclass identification and reactivity specificity of mAb 4H3

**Figure 2:**
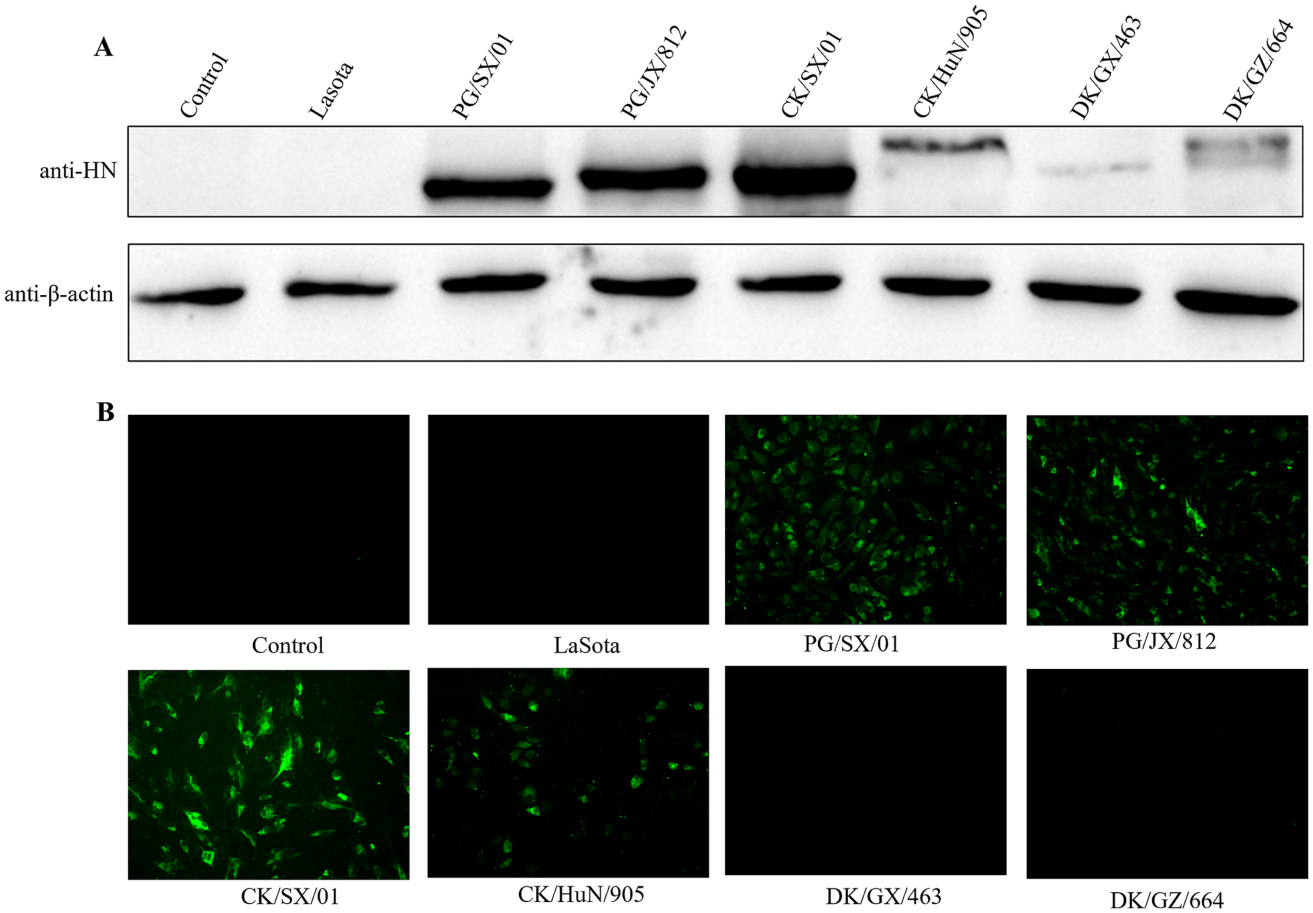
Specificity analysis of mAb 4H3 for binding to diverse PPMV-1 and NDV strains. **(A)** Western blot analysis of mAb 4H3. DF-1 cells were infected with PPMV-1 strains (PG/SX/01, PG/JX/812), chicken-derived NDV strains (CK/SX/01, CK/HuN/905), duck-derived NDV strains (DK/GX/463, DK/GZ/664), and the NDV vaccine strain Lasota at an MOI of 1. At 12 hpi, cells were lysed and supernatants were collected and subjected to SDS-PAGE and western blot analysis. Uninfected DF-1 cells served as the negative control, and *β*-actin was used as the internal control. **(B)** Immunofluorescence assay (IFA) analysis of mAb 4H3. DF-1 cells were infected with the indicated viruses at an MOI of 1. At 12 hpi, cells were fixed with 4% paraformaldehyde and permeabilized with 0.5% Triton X-100. Subsequently, IFA was performed using purified mAb 4H3 as the primary antibody and fluorescein isothiocyanate (FITC)-conjugated goat anti-mouse IgG as the secondary antibody. Green fluorescence indicates the specific binding of mAb 4H3 to the HN protein in PPMV-1- or NDV-infected DF-1 cells.

Subclass identification indicated that mAb 4H3 was classified as the IgG2a subclass with a *κ* light chain (Table 1). Western blot analysis showed that mAb 4H3 specifically binds to the HN protein of PPMV-1 strains (PG/SX/01, PG/JX/812) and chicken-derived NDV strains (CK/SX/01, CK/HuN/905), yielding a distinct band at approximately 75 kDa—consistent with the molecular weight of the native HN protein. In contrast, no specific immunoreactive bands were observed for the NDV vaccine strain Lasota, whereas only extremely weak immunoreactive bands were detectable in samples of the duck-derived NDV strains DK/GX/463 and DK/GZ/664 (Figure 2A).

**Table 1:** Identification of the subclasses of mAb 4H3.

OD_450_	0.032	0.041	0.669	0.057	0.082	0.05	0.301	0.031
Subclass	IgM	IgG1	**IgG2a**	IgG2b	IgG3	IgA	***κ***	*λ*

The bold values meet the criteria for a positive determination.

IFA results were in agreement with Western blot findings: robust green fluorescence was detected in DF-1 cells infected with PPMV-1 strains and chicken-derived NDV strains, whereas no fluorescent signals were observed in cells infected with the other tested viruses or the negative control (Figure 2B). Collectively, these results confirm that mAb 4H3 exhibits high specificity for PPMV-1 strains.

### 3.3 Epitope mapping of mAb 4H3

**Figure 3:**
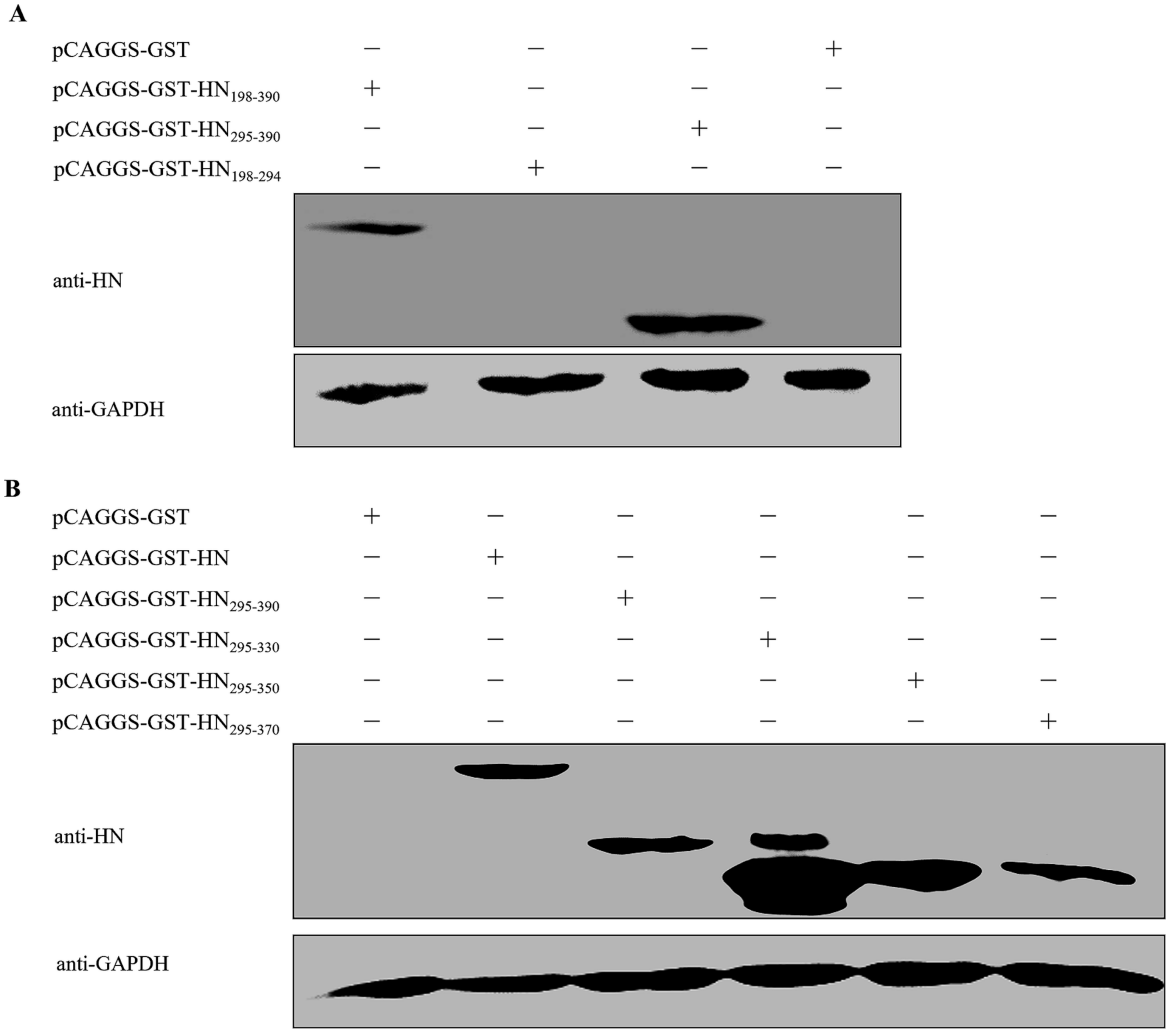
Mapping of the critical region of the HN protein recognized by mAb 4H3. **(A)** Identification of the HN protein-binding region of mAb 4H3. The HN_198–390_ fragment was split into two non-overlapping fragments (HN_198–294_ and HN_295–390_), which were separately cloned into the pCAGGS-GST eukaryotic expression vector, generating recombinant plasmids pCAGGS-GST-HN_198–294_ and pCAGGS-GST-HN_295–390_. After transfection into HEK293T cells, cells were lysed at 48 h post-transfection, and western blot analysis was performed to identify the critical region of the HN protein specifically recognized by mAb 4H3. **(B)** Detection of C-terminal truncation of HN_295–390_. The HN_295–390_ fragment was C-terminally truncated to generate a panel of derivatives (HN_295–330_, HN_295–350_, HN_295–370_), which were cloned into the pCAGGS-GST vector and transfected into HEK293T cells. Western blot analysis was then used to evaluate mAb 4H3’s specific binding reactivity to these truncation derivatives.

To delineate the epitope recognized by mAb 4H3, the HN_198–390_ aa region was initially divided into two non-overlapping fragments: HN_198–294_ and HN_295–390_. Western blot analysis indicated that mAb 4H3 specifically bound to HN_295–390_ but not HN_198–294_, localizing the epitope to the HN_295–390_ aa domain (Figure 3A).

For subsequent epitope refinement, a panel of C-terminally truncated derivatives of HN_295–390_ was constructed, including HN_295–330_, HN_295–350_, and HN_295–370_. Western blot analysis demonstrated that mAb 4H3 displayed specific reactivity with all three truncation derivatives, indicating the epitope recognized by mAb 4H3 was localized to the 295–330 aa N-terminal subregion of HN_295–390_ (Figure 3B).

**Figure 4:**
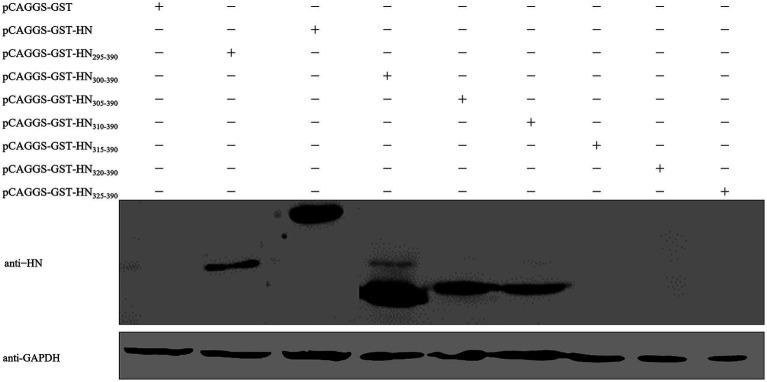
Mapping of the critical epitope of the HN protein recognized by mAb 4H3. Based on the localization of the mAb 4H3-reactive critical region to the N-terminal segment of HN_295–390_, a panel of N-terminally truncated derivatives of HN_295–390_ was generated with sequential 5-amino acid truncations at the N-terminus, yielding HN_300–390_, HN_305–390_, HN_310–390_, HN_315–390_, HN_320–390_, and HN_325–390_. Expression plasmids encoding these N-terminally truncated mutants were transfected into HEK293T cells, and western blot analysis was performed to define the critical epitope recognized by mAb 4H3.

To precisely define the minimal linear epitope recognized by mAb 4H3, N-terminally stepwise truncated mutants of HN_295–330_ (5 amino acids per truncation) were generated: yielding HN_300–390_, HN_305–390_, HN_310–390_, HN_315–390_, HN_320–390_, and HN_325–390_. The recombinant plasmids encoding these truncation derivatives were transfected into HEK293T cells, and Western blot analysis verified that mAb 4H3 specifically recognized HN_300–390_, HN_305–390_, and HN_310–390_, but failed to bind HN_315–390_, HN_320–390_, or HN_325–390_ (Figure 4). Taken together, the present data demonstrate that the linear epitope of the PPMV-1 HN protein recognized by mAb 4H3 maps to amino acid residues 310–314, namely ^310^DRVWF^314^.

### 3.4 Homology and structural analysis of the identified epitope

**Figure 5:**
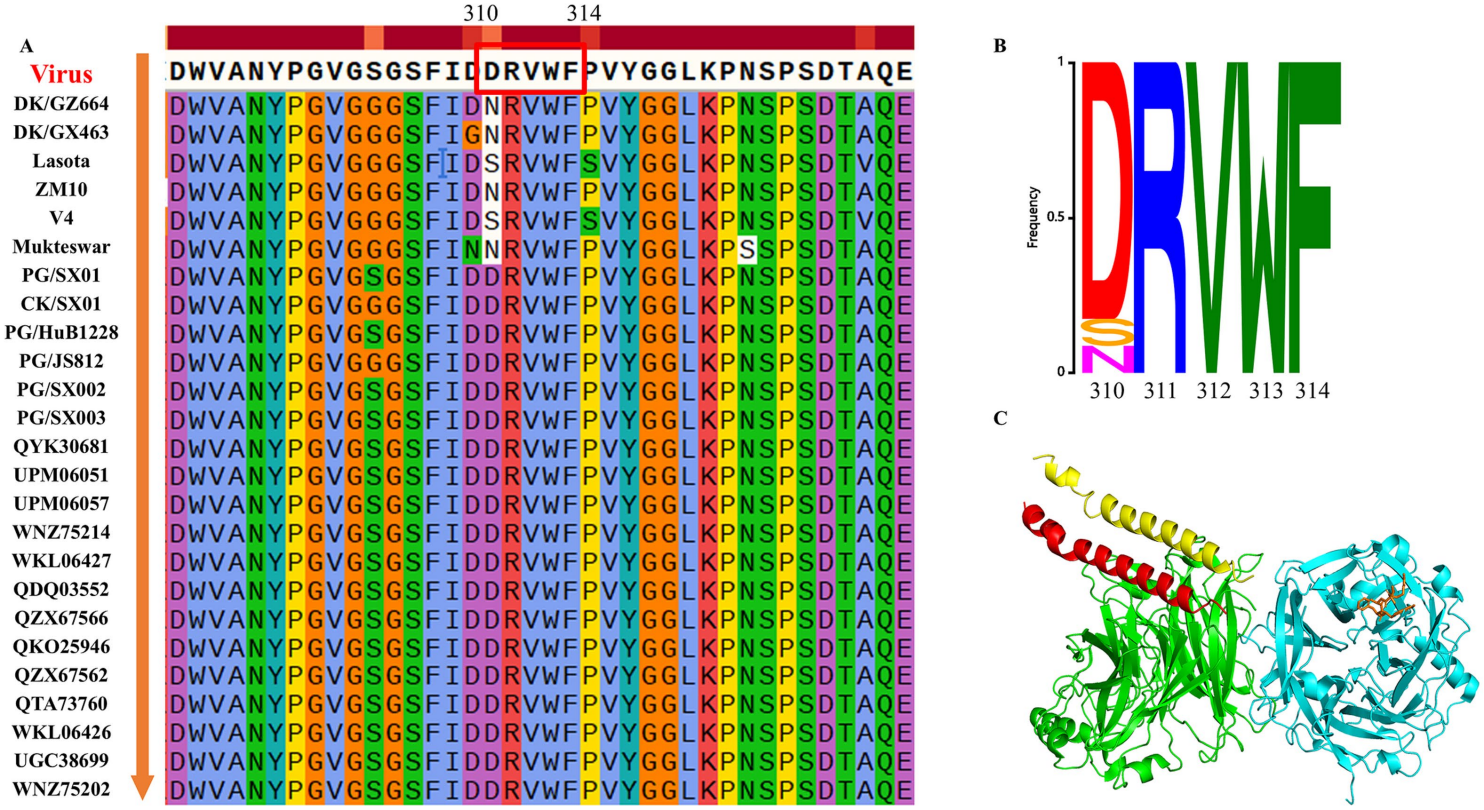
Conservation analysis and structural modeling of the identified epitope in the HN protein. **(A)** Amino acid sequence differences of the HN protein among NDV vaccine strains, pigeon-derived PPMV-1, and NDV strains from diverse sources were visualized using SnapGene. The B-cell epitope recognized by mAb 4H3 is marked with a red box. The epitope ^310^DRVWF^314^ is highly conserved among PPMV-1 but harbors amino acid substitutions in NDV vaccine strains. **(B)** Conservation of the mAb 4H3-recognized B-cell epitope between NDV vaccine strains, PPMV-1 strains and NDV strains from diverse sources was visualized using WeMol. **(C)** Three-dimensional (3D) structure of the HN protein. The B-cell epitope recognized by mAb 4H3 is highlighted in a stick model. The structural model was visualized with PyMOL3.1, with the crystal structure of the NDV HN protein (PDB ID: 3T1E) used as the template.

Sequence alignment of PPMV-1 and NDV strains across different hosts and genotypes revealed that the epitope ^310^DRVWF^314^ is highly conserved among PPMV-1 strains (Figures 5A,B). In contrast, distinct amino acid substitutions at position 310 of the HN protein (D310N or D310S) were identified in NDV vaccine strains (e.g., Lasota, V4) and duck-derived NDV, which underscores the critical role of this residue in the specific binding of mAb 4H3 to the PPMV-1 HN protein. Notably, these substitutions were strain-dependent: the vaccine strains LaSota and V4 carried the D310S mutation, whereas the Mukteswar and ZM10 strains, along with duck-derived isolates DK/GX/463 and DK/GZ/664, harbored D310N. This observation may explain the complete absence of specific immune reactivity of mAb 4H3 against NDV vaccine strains and its relatively weak specific immune reactivity against duck-derived NDV. Furthermore, structural modeling of the HN protein (based on PDB: 3T1E) localized the ^310^DRVWF^314^ epitope to the neuraminidase (NA) globular domain (Figure 5C). This conserved epitope is critical for maintaining the conformational stability of the NA globular domain and facilitating its dimerization of the HN protein.

## 4 Discussion

PPMV-1 is a major pathogen threatening the pigeon industry, and the lack of specific diagnostic tools and effective vaccines has hindered PND control (Zou et al., 2022). The HN protein of PPMV-1 is a key antigen involved in viral pathogenesis and immune response, rendering it an ideal target for the detection of PPMV-1 pathogens and specific antibodies (Li et al., 2015; Muzyka et al., 2014). In this study, we successfully generated a specific mAb 4H3 against the HN protein of PPMV-1 and identified its recognized epitope, providing valuable insights for PND diagnosis and vaccine development.

The HN protein plays multiple pivotal roles in viral entry and egress, encompassing binding to sialic acid receptors, activating the fusion (F) protein to mediate membrane fusion and viral invasion, and cleaving sialic acid residues to facilitate the release of progeny virions (Cheng et al., 2016; Snoeck et al., 2013; Welch et al., 2014; Hurley et al., 2023). HN consists of an N-terminal transmembrane domain, a stalk region, and an enzymatically active NA domain (Yuan et al., 2011). The recombinant HN protein (198–390 aa) used as an immunogen was selected based on sequence conservation and antigenicity analysis (Supplementary Figures S1–S3). This region is highly conserved among PPMV-1 strains, ensuring that the generated antibody can recognize multiple PPMV-1 isolates. The prokaryotic expression system was chosen for its high efficiency and low cost, and the purified protein exhibited high purity and immunogenicity (Xing et al., 2022; Wang et al., 2022), as evidenced by the high titer of specific antibodies in immunized mice.

The mAb 4H3 exhibits high specificity for PPMV-1 strains, showing no reactivity with the Lasota vaccine strain, no or very weak cross-reactivity with duck-derived NDV, and no cross-reactivity with avian influenza virus (AIV) or infectious bronchitis virus (IBV) (data not shown). This distinct specificity renders it well-suited for the development of PPMV-1-specific diagnostic assays, effectively mitigating false positives arising from cross-reactivity with other avian viral pathogens. Here, the PPMV-1 HN protein was expressed in *E. coli* as inclusion bodies. Unlike native HN proteins, which require extensive glycosylation and chaperone-assisted folding to achieve their functional tetrameric state and biological activities (hemagglutination, neuraminidase, and fusion promotion), proteins produced in prokaryotic systems lack these modifications. This leads to misfolded aggregates characterized by mispaired disulfide bonds and the absence of glycosylation. Such profound structural abnormalities may distort or obscure conformational epitopes, thereby compromising the accuracy of subsequent epitope mapping and related functional analyses. To mitigate this potential interference and ensure the authenticity of identified epitopes, subsequent epitope mapping experiments were performed using eukaryotic expression systems (HEK293T cells). This strategic combination leverages the high expression efficiency of prokaryotic systems for immunogen preparation, while utilizing the advantages of eukaryotic systems in correctly folding protein fragments and mimicking native post-translational modifications. Collectively, this approach significantly enhances the accuracy and reliability of epitope identification, laying a solid foundation for further investigations into the antigenic characteristics and immunogenicity of PPMV-1 HN protein. Epitope mapping identified that mAb 4H3 targets the minimal epitope ^310^DRVWF^314^, which is highly conserved across PPMV-1 strains and chicken-derived Class II NDV strains—suggesting a pivotal role in sustaining viral structure and function. Notably, amino acid substitutions at position 310 of the HN protein in NDV vaccine strain Lasota and duck-derived NDV may represent a key determinant impairing the binding of mAb 4H3 to its target epitope. Furthermore, structural localization (based on PDB: 3T1E) places the ^310^DRVWF^314^ epitope within the NA globular domain of the HN protein, where it is involved in sustaining the NA domain’s conformational stability and promoting its dimerization. Prior to structural modeling, sequence alignment was performed between PPMV-1 and the NDV Australia-Victoria strain—the reference strain whose HN protein structure was originally resolved. The analysis yielded a high sequence identity of over 88%. Given that PPMV-1 represents an antigenic lineage that diverged from ancestral NDV via host-specific adaptation in pigeons, the NDV HN structure provides a biologically rigorous template for our structural localization analysis (Yuan et al., 2011; Ruan et al., 2020). This epitope potentially contributes to sialic acid receptor binding, NA enzymatic activity, and F protein activation regulation, while embodying the application potential of an immunodominant epitope. Future studies should validate whether this epitope functions as a neutralizing epitope and explore the impact of amino acid mutations on its antigenicity. It is worth noting that the truncation sites in this study were selected primarily to ensure comprehensive sequence coverage. Although this approach successfully identified the ^310^DRVWF^314^ motif, the potential impact of “blind truncation” on protein conformational stability cannot be overlooked. Future designs would benefit from integrating structural bioinformatic tools to predict domain boundaries before fragment construction. Such a strategy would prevent the inadvertent cleavage of essential structural elements, ensuring that truncated proteins better mimic the native conformation and reducing the likelihood of yielding non-reactive, misfolded expression products.

Given the robust reactivity of mAb 4H3 with PPMV-1 observed in both IFA and Western blot assays, the mAb 4H3 generated in this study may be exploited for the specific detection of PPMV-1. For instance, it can be applied to IFA or immunochromatographic colloidal gold test strips, thereby enabling rapid and specific diagnosis of PPMV-1 infection. It also holds promise as a valuable tool for investigating HN protein function, antigen localization, and the viral pathogenesis of PPMV-1. Furthermore, the identified linear epitope ^310^DRVWF^314^ may be instrumental in the development of PPMV-1 epitope-based vaccines. Epitope-based vaccines offer distinct advantages over traditional vaccine platforms, including high specificity, low toxicity, and superior stability (Tan et al., 2022; Tan et al., 2024; Adam et al., 2023). The identification of the ^310^DRVWF^314^ epitope opens several critical avenues for future research. Primarily, viral neutralization assays will be conducted to evaluate the biological potency of mAb 4H3 and its ability to inhibit viral entry. To refine the molecular map of the epitope, site-directed mutagenesis will be employed to delineate the energetic contributions of individual residues to the binding interface. Moreover, to resolve the high-resolution three-dimensional structure of the mAb-antigen complex using X-ray crystallography or Cryo-EM, which will yield definitive insights into the spatial orientation and stoichiometry of the interaction. Nonetheless, several limitations of the present study should be acknowledged. First, the neutralizing capacity of mAb 4H3 has not yet been validated in animal models. Second, the reliance on truncated fragments for epitope mapping necessitates further verification at the single-residue level. Third, the broad-spectrum binding activity of this mAb remains to be confirmed against a more diverse collection of field isolates from different lineages and regions. Lastly, the binding kinetics between mAb 4H3 and the HN protein have yet to be quantified. Subsequent investigations utilizing surface plasmon resonance (SPR) are warranted to provide a more rigorous characterization of these molecular interactions.

## 5 Conclusion

In conclusion, we successfully generated a specific mAb 4H3 against the HN protein of PPMV-1 and mapped its target epitope to ^310^DRVWF^314^. This mAb 4H3 exhibits high specificity for PPMV-1, showing no reactivity with the Lasota vaccine strain, which has the potential to be developed as a valuable tool for the detection and diagnosis of PPMV-1 strains.

## Data Availability

The datasets presented in this study can be found in online repositories. The names of the repository/repositories and accession number(s) can be found in the article/Supplementary material.

## Footnotes

1
https://wemol.wecomput.com/ui/
