# Supplementary material for: Generation, characterization and epitope mapping of monoclonal antibody 4H3 specific to the HN protein of pigeon paramyxovirus type 1 (PPMV-1)
Source: Front Microbiol. 2026 Apr 24;17:1808407. doi: 10.3389/fmicb.2026.1808407 (PMC13152863; doi:10.3389/fmicb.2026.1808407)
Supplement: Supplementary file 1 [file Data_Sheet_1.DOCX]

Supplementary Material

Generation, characterization and epitope mapping of monoclonal antibody 4H3 specific to the HN protein of pigeon paramyxovirus type 1 (PPMV-1)

Xu Wang¹, Hongfeng Ren¹, Pei Li¹, Zhonglin Huang¹, Xing Li¹, Yang Wang¹^,2^, Yang Li^2^, Libin Liang^1^*, Junping Li^1^*

¹Shanxi Key Laboratory of Animal Disease Research, Prevention and Control, College of Veterinary Medicine, Shanxi Agricultural University, Jinzhong, China;

²China Animal Health and Epidemiology Center, Qingdao, Shandong 266032, China

*Correspondence:

Libin Liang, [lianglibin@sxau.edu.cn](mailto:lianglibin@sxau.edu.cn); Tel.: +86-354-6285988

Junping Li, [lijunping@sxau.edu.cn](mailto:lijunping@sxau.edu.cn); Tel: +86-354-6285988

## Supplementary Figures


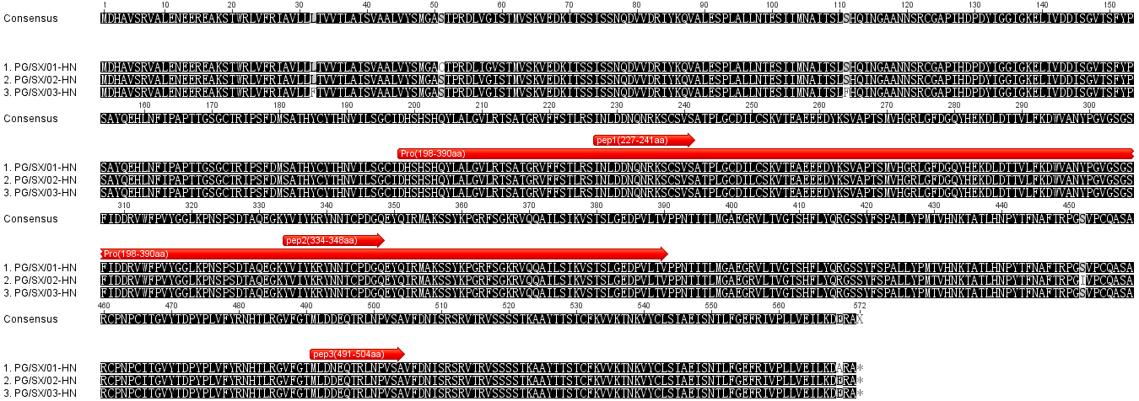


**Supplementary Figure 1.** Sequence alignment of the HN protein from three PPMV-1 strains isolated in our laboratory. The amino acid sequences of the HN protein from three pigeon-derived PPMV-1 strains were analyzed using Geneious software to evaluate sequence homology, which revealed that the 198–390 aa region had the highest conservation.


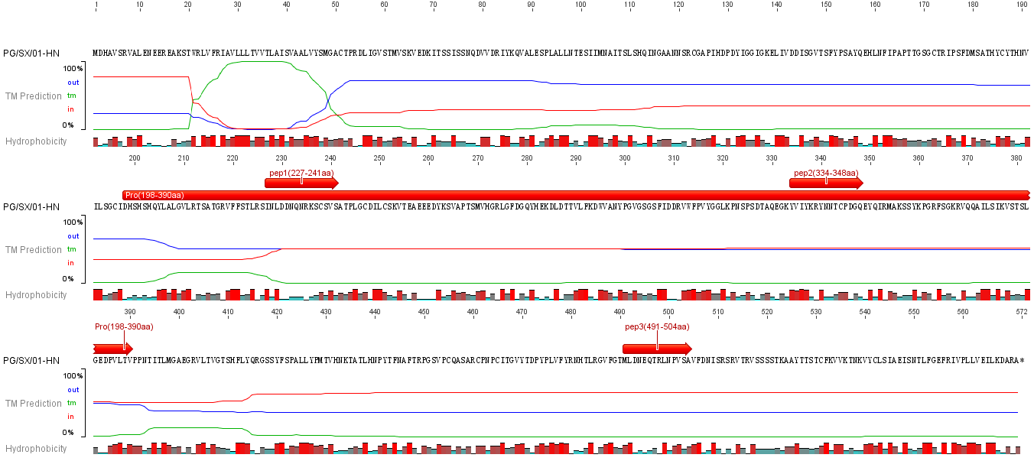


**Supplementary Figure 2.** Secondary structure analysis of the HN protein from PPMV-1 strain PG/SX/01. The amino acid sequence was analyzed with Geneious software, including transmembrane (TM) prediction for the membrane protein and hydrophobicity prediction for the hydrophobicity profile; the 198–390 aa region exhibited low hydrophobicity.


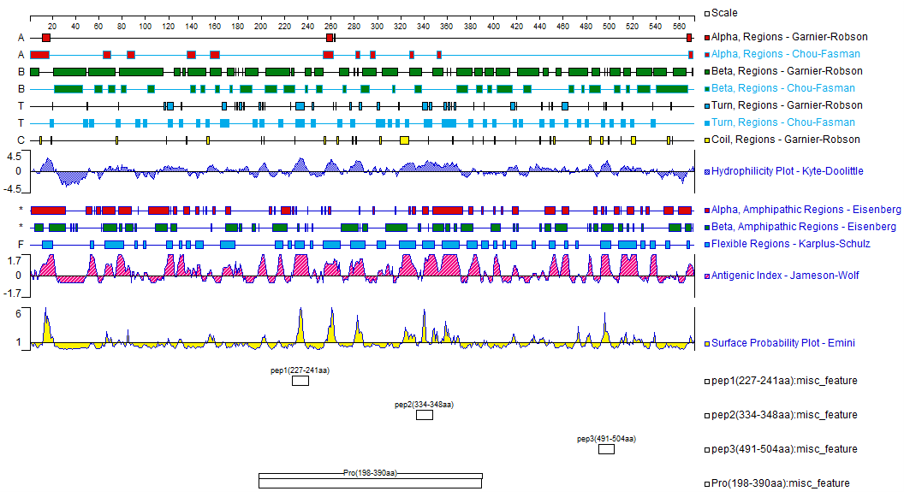


**Supplementary Figure 3.** Antigenicity and secondary structure analysis of the HN₁₉₈₋₃₉₀ region. The secondary structural domains, antigenicity, and surface probability of the protein were analyzed using the Protean program embedded in the DNASTAR software package.
